# Physiological Signaling and Structure of the HGF Receptor MET

**DOI:** 10.3390/biomedicines3010001

**Published:** 2014-12-31

**Authors:** Gianluca Baldanzi, Andrea Graziani

**Affiliations:** 1 Department Translational Medicine, University Piemonte Orientale, via Solaroli 17, 28100 Novara, Italy; 2 Università Vita-Salute San Raffaele, via Olgettina 58, 20132 Milano, Italy; E-Mail: andrea.graziani@med.unipmn.it

**Keywords:** signaling pathways, tyrosine kinase receptor, protein–protein interaction, SH2 domain, post translational modification, signal transduction

## Abstract

The “hepatocyte growth factor” also known as “scatter factor”, is a multifunctional cytokine with the peculiar ability of simultaneously triggering epithelial cell proliferation, movement and survival. The combination of those proprieties results in the induction of an epithelial to mesenchymal transition in target cells, fundamental for embryogenesis but also exploited by tumor cells during metastatization. The hepatocyte growth factor receptor, MET, is a proto-oncogene and a prototypical transmembrane tyrosine kinase receptor. Inhere we discuss the MET molecular structure and the hepatocyte growth factor driven physiological signaling which coordinates epithelial proliferation, motility and morphogenesis.

## 1. Background Introduction

The Hepatocyte Growth Factor (HGF) was originally identified as a soluble factor promoting hepatocyte growth and liver regeneration [1]. In a parallel way a Scatter Factor (SF) was identified as cytokine secreted by fibroblast promoting dissociation and motility of epithelial cells in culture [2].

Molecular cloning demonstrated that HGF and SF are the same growth factor produced by cells of mesenchymal origin and promoting migration and proliferation depending on the epithelial cell targeted [3]. The HGF receptor (MET) is a prototypal tyrosine kinase receptor which plays a key role in the interaction between mesenchyme and epithelia during embryogenesis and tissue homeostasis [4]. The observation that MET is a proto-oncogene and that its signaling is often subverted in cancer has promoted deep investigations on its molecular structure and signaling proprieties which are reviewed herein.

## 2. HGF

The *HGF* gene map to 7q21.11, in the same chromosomal region of its receptor MET. By alternative splicing the gene give rise to several transcripts beside the full length isoform (728 aa corresponding to 83 kDa, increased to 92 kDa after glycosylation) [5]. The full length pre-pro-HGF feature a strong sequence and structural homology to plasminogen [6,7,8], presenting a signal peptide for secretion (residues 1–31), an amino-terminal heparin binding domain (residues 37–123), 4 kringle domains (residues 128–206, 211–288, 305–383 and 391–469) and a serine protease-like domain (residues 495–721) [5,9,10]. A close homolog of HGF is macrophage stimulating protein (MSP) with a 45% identity and a similar organization with 4 kringle domains [11].

Two other HGF splicing isoforms have been extensively characterized consisting of the amino-terminal domain linked in tandem with, respectively, the first one (NK1, 24 kDa) or the first two (NK2, 36 kDa) kringle domains [12]. All three isoforms bind to the receptor tyrosine kinase MET and evoke signaling but with different affinity and potency [13]. Indeed, NK1 stimulates cell proliferation, migration and tubular morphogenesis, though at reduced potency and with greater heparan sulfate (HS) dependence compared to full-length HGF, suggesting that the primary MET binding site is contained within this fragment [14]. Conversely, NK2 acts as a competitive inhibitor of full length HGF promoted mitogenicity, but retains motogenic activity *in vitro* and *in vivo* [15,16].

All HGF isoforms are synthesized as pre-peptides that undergo proteolytic cleavage at residue 31 to remove the leader sequence. Full-length pro-HGF also undergoes extensive glycosylation (*N*-linked at residue 294, 402, 566 and 653, *O*-linked at residue 476), which is dispensable for biological activity but promotes secretion [17,18]. Full length HGF requires proteolytic cleavage at the beginning of the serine protease like domain (between R_494_ and V_495_) to become a biologically active heterodimer consisting of disulfide-linked α (residues 32–494, 69 kDa after glycosylation) and β (residues 495–728, 32–34 kDa depending on the extension of glycosylation) chains [19,20,21]. Several serine proteases in serum are capable of HGF activation *in vitro*, including urokinase-type plasminogen activator and tissue-type plasminogen activator [22], HGF activator [23,24], matriptase [25], hepsin [26] and blood factors XIa and XIIa [24]. Extracellular processing may play a regulatory role on HGF biological activity as localized activation of pro-HGF has been evidenced in injured tissues [27].

The amino-terminal HS binding domain and secondary binding sites in the first kringle domain [28] play a major role in HGF biology representing a high capacity, medium affinity binding site in many cell types, comprising those unresponsive to HGF stimulation [29]. Those binding sites are constituted by heparan and dermatan sulfates found on extracellular matrix proteins such as decorin, syndecans and biglycan [30,31]. HGF also specifically binds to sulfoglycolipids which may also modulate its activity [32]. The abundance of HGF binding sites in extracellular matrix and basal membranes allow the local accumulation of HGF and its release in a spatially and temporally restricted manner through matrix turnover [31]. Furthermore HGF binding to cell-surface HS increase local HGF concentrations and putatively may allow for HGF dimerization [33] or change in conformation [30], effectively promoting receptor clustering and kinase activation [34,35]. Furthermore HS-modified CD44v3 interacts with MET and increase HGF-induced signal transduction [36]. Conversely, HS interactions with MET are substantially weaker than those with HGF, and their contribution to the stability a ternary HGF-HS-MET complex may not be critical for all HGF responses [37,38].

Lai and Goldschneider [39] reported a naturally occurring hybrid cytokine consisting of the HGF β chain and IL-17 acting as a pre-pro-B cell growth-stimulating factor. Although the molecular mechanisms by which such cytokine is produced are still uncharacterized it represents an efficient way to coordinate signals through both IL-7R and MET promoting B cell development.

## 3. MET

### 3.1. Gene and Transcript 

The *MET* gene is located on 7q31 and encodes a 1390 aa protein, with an apparent molecular weight of 190 kDa, which is the most abundant form in a variety of tissues and cell lines. The use of an alternative in-frame splice junction results in a longer transcript variant with 18 additional amino acids in the extracellular region (1408 aa, apparent molecular weight of 170 kDa) [40]. Alternative splicing originates also a variant transcript of MET lacking 47 amino acids in the juxtamembrane region of the cytoplasmic domain present in adult mouse tissues including kidney, liver, and brain at lower levels than the full-length transcript. The deleted region in the cytoplasmic domain contains the S_985_ phosphorylated by protein kinase C down-regulating of MET kinase activity [41].

Truncated forms, resulting from receptor shedding, were reported in blood and in tumor derived cell lines, those forms are putatively involved in carcinogenesis [42,43,44,45]. Conversely, in stress conditions, the MET receptor is cleaved by caspases at D_1002_ within its juxtamembrane region, generating a pro-apoptotic intracellular fragment of 40 kDa [46].

MET is predominantly expressed in epithelial derived cells such as the epithelial layer lining the gastrointestinal tract, liver kidney, thyroid and in keratinocytes [47,48]. Lower levels were detected in other cell types such as endothelial cells [49], hematopoietic progenitors [50], B cells [51] and the brain [48]. Conversely, HGF is produced by several mesenchymal cells but is also contained in platelet granules and released during aggregation promoting wound closure and epithelial cell proliferation [5]. Thus the HGF-MET pair are at the base of epithelial mesenchymal interaction during embryogenesis, wound closure and angiogenesis [52].

### 3.2. MET Structure 

**Figure 1:**
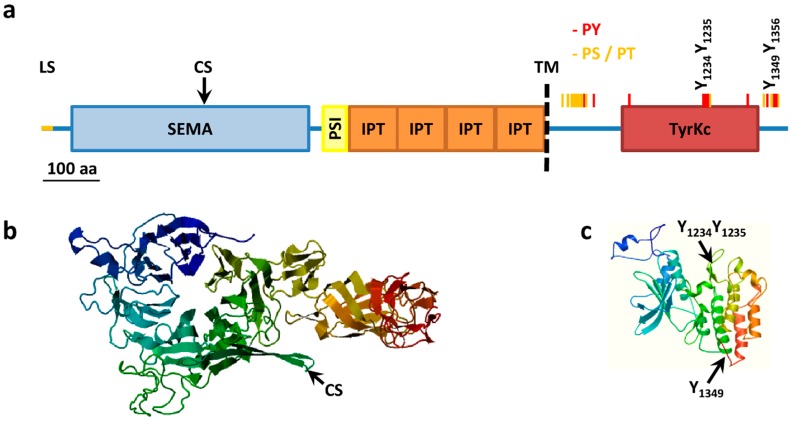
MET structure. (**a**) MET structural domains. MET domains as predicted using SMART. SEMA, Semaphorin domain (aa 52–496); PSI, Plexin semaphorin domain (aa 519–562); IPT, IG like plexins transcription factor (aa 562–655); IPT, IG like plexins transcription factor (aa 656–739); IPT, IG like plexins transcription factor (aa 741–836); IPT, IG like plexins transcription factor (aa 838–934); TM, transmembrane region (aa 933–955); TyrKc, tyrosine kinase (aa 1078–1337); Further features specified: LS, Leader sequence (aa 1–24); CS, Cleavage site (aa 307–308); PY, tyrosine phosphorylation sites (red bars); PS/PT, serine/threonine phosphorylation sites (yellow bars); (**b**) Model of extracellular portions of MET from the SWISS-MODEL repository aa 42 to 741, Model_id ee0753c4f68e188bddb0c66890beee23_UP000052_4; (**c**) Model of intracellular portions of MET from the SWISS-MODEL repository, aa 1024 to 1352, Model_id ee0753c4f68e188bddb0c66890beee23_UP000052_3.

During synthesis in the endoplasmic reticulum the leader sequence (aa 1–24) is removed and MET is extensively co-translationally glycosylated at residue 106 [53] (further putative *N*-linked glycosylation sites: 45, 149, 202, 399, 405, 607, 635, 785, 879 and 930) to give a 190 kDa single chain precursor with several disulfide bonds [8,54,55]. Glycosylation and folding are a requisite for the following cleavage between residue 307 and 308 to give a mature heterodimer composed of an extracellular 50 kDa α-chain and a transmembrane 140 kDa β-chain, linked together by disulfide bridges. Cleavage is carried out by furin proteases but is not required for receptor activation by HGF [56], indeed the longer transcript variant is exposed on cell surface and phosphorylated but is not processed in an α–β heterodimer [40]. The extracellular α-chain contains the ligand binding pocket while the intracellular portion of the β-chain contains the tyrosine kinase domain and a conserved two-tyrosine multifunctional docking site that interacts with multiple SRC homology 2 (SH2) containing intracellular signal transducers (Figure 1).

Paralleling the similarity between HGF and MSP, the closest homolog of MET is the MSP tyrosine kinase receptor RON, with an overall identity of 33% of amino acids, mainly at the level of the tyrosine kinase domain with a 64% identity [57]. Conversely the extracellular portion presents a series of domains with homology to the semaphorin receptor family (semaphorin domain, SEMA), to plexins and integrins (Plexin SEMAphorins Integrines domain, PSI) and four immunoglobulin-like folds shared by plexins and transcription factors (IPT domain). MET structure and key post-translational modification are summarized in Figure 1.

### 3.3. MET Activation and Signaling

The tyrosine kinase activity of MET transduces the mitogenic and motogenic signals elicited by HGF [58,59]. Notably, beside HGF, the *Listeria monocytogenes* surface protein InlB is a MET agonist that mimics HGF signaling, inducing bacterial entry through exploitation of a host RTK signaling pathway [60]. While a crystal structure of mature α/β HGF bond to MET is still missing, several studies employed HGF fragments and mutational analysis to identify the contact interface. A direct interaction between HGF β-chain and MET SEMA domain emerges from crystallographic studies but is insufficient to promote receptor activation [7,8]. Conversely, the N terminal HGF region corresponding to NK1 induces signaling by high affinity binding to MET SEMA domain [35,38,61], although other authors reported an interaction of this region with MET IPT domains 3 and 4 [62]. Ligand binding give rise to a change of MET conformation allowing:

(1) dimerization, which is not only promoted by tendency of self-association of the HGF ligand [33,35] but also of MET SEMA domains [63];
(2) auto-phosphorylation on the activation loop (Y_1230_DKEY_1234_Y_1235_) on Y_1234_ and Y_1235_ which is essential for biological activity and phosphorylation of substrates [64,65,66];
(3) auto-phosphorylation of the *C* terminal Y_1349_ and Y_1356_ tyrosine residues. Those tyrosines represent a docking site for downstream effectors [67,68], while in the resting state the non-phosphorylated tail acts as an intramolecular inhibitor of the kinase activity [69].

The key event inducing signaling is the auto-phosphorylation of the multifunctional docking site made of the tandem arranged Y_1349_ and Y_1356_ in the degenerate sequence YVH/NV (Table 1). Those two residues mediate intermediate- to high-affinity interactions with multiple SH2 containing signal transducers. Indeed the mutation of the two tyrosines results in loss of biological function [67,68]. In particular a pivotal role is played by Y_1356_, as MET containing an Y_1356_F substitution is unable to transduce motogenic signals. Y_1356_VNV represents a consensus binding site for multiple effectors such as the adaptor GRB2, the p85 subunit of phosphatidyl inositol 3-kinase (PI3K), phospholipase Cγ (PLCγ), and the tyrosine-protein phosphatase SHP-2 [68,70,71,72]. Together with Y_1349_ this tyrosine is also required for association and phosphorylation of the adaptor SHC [73]. *In vivo*, mutation of both residues in the mouse genome caused embryonic lethality, with placenta, liver, and limb muscle defects, mimicking the phenotype of MET null mutants [74].

A fundamental amplification role in the MET signalosome assembly is played by the Y_1356_ mediated recruitment of the GRB2-associated binding protein (GAB1). Indeed selective disruption of the GRB2 consensus in MET impairs GRB2 and GAB1 association resulting in decreased mitogen-activated protein kinase (MAPK) activation. This reduced level of signaling is sufficient for motility but not for branching morphogenesis and cell transformation [70,75]. *In vivo*, disrupting the consensus for GRB2 binding allows mice development to proceed to term without affecting placenta and liver, but caused a striking reduction in limb muscle coupled to a generalized deficit of secondary fibers [74]. Conversely, a point mutation which duplicates the GRB2 binding site, super-activates the Ras GTPase pathway and prevents the binding of the other intracellular transducers. This increased the transforming ability of the oncogene but abolished its metastatic potential [76].

**Table 1:** MET phosphorylation sites.

SS	MS	aa	*Homo sapiens*	aa	*Mus musculus*	aa	*Rattus norvegicus*
0	6	S966-p	KQIkDLGsELVRyDA	S964	RKHKDLGSELVRYDA	S967	RKHKDLGSELVRYDA
0	5	Y971-p	LGsELVRyDARVHtP	Y969	LGSELVRYDARVHtP	Y972	LGSELVRYDARVHTP
0	6	T977-p	RyDARVHtPHLDRLV	T975-p	RYDARVHtPHLDRLV	T978	RYDARVHTPHLDRLV
6	0	S985-p	PHLDRLVsARsVsPt	S983-p	PHLDRLVsARSVsPT	S986-p	PHLDRLVsARSVSPT
0	19	S988-p	DRLVsARsVsPttEM	S986	DRLVsARSVsPTTEM	S989	DRLVsARSVSPTTEM
0	32	S990-p	LVsARsVsPttEMVs	S988-p	LVsARSVsPTTEMVs	S991	LVsARSVSPTTEMVS
0	11	T992-p	sARsVsPttEMVsNE	T990	sARSVsPTTEMVsNE	T993	sARSVSPTTEMVSNE
0	7	T993-p	ARsVsPttEMVsNEs	T991	ARSVsPTTEMVsNEs	T994	ARSVSPTTEMVSNES
0	20	S997-p	sPttEMVsNEsVDyR	S995-p	sPTTEMVsNEsVDyR	S998	SPTTEMVSNESVDYR
1	43	S1000-p	tEMVsNEsVDyRAtF	S998-p	TEMVsNEsVDyRATF	S1001	TEMVSNESVDYRATF
11	361	Y1003-p	VsNEsVDyRAtFPED	Y1001-p	VsNEsVDyRATFPED	Y1004	VSNESVDYRATFPED
0	9	T1006-p	EsVDyRAtFPEDQFP	T1004	EsVDyRATFPEDQFP	T1007	ESVDYRATFPEDQFP
0	15	Y1026-p	GsCRQVQyPLTDMSP	Y1024	GACRQVQYPLTDLSP	Y1027	GACRQVQYLLTDLSP
0	31	Y1093-p	RGHFGCVyHGtLLDN	Y1091	RGHFGCVYHGTLLDN	Y1094	RGHFGCVYHGTLLDS
4	112	Y1230-p	FGLARDMyDkEyysV	Y1228-p	FGLArDMyDKEyysV	Y1231	FGLARDMYDKEyySV
39 *	735	Y1234-p	RDMyDkEyysVHNkt	Y1232-p	rDMyDKEyysVHNKt	Y1235-p	RDMYDKEyySVHNKT
38 *	443	Y1235-p	DMyDkEyysVHNktG	Y1233-p	DMyDKEyysVHNKtG	Y1236-p	DMYDKEyySVHNKTG
1	177	S1236-p	MyDkEyysVHNktGA	S1234-p	MyDKEyysVHNKtGA	S1237	MYDKEyySVHNKTGA
0	5	T1241-p	yysVHNktGAKLPVK	T1239-p	yysVHNKtGAKLPVK	T1242	yySVHNKTGAKLPVK
6	5	Y1313-p	EyCPDPLyEVMLkCW	Y1311-p	EYCPDALyEVMLKCW	Y1314	EYCPDALYEVMLKCW
0	6	T1343-p	RISAIFstFIGEHyV	T1341	RISSIFSTFIGEHyV	T1344	RISSIFSTFIGEHYV
24 *	122	Y1349-p	stFIGEHyVHVNAty	Y1347-p	STFIGEHyVHVNATy	Y1350	STFIGEHYVHVNATY
0	40	T1355-p	HyVHVNAtyVNVKCV	T1353	HyVHVNATyVNVKCV	T1356	HYVHVNATYVNVKCV
22 *	120	Y1356-p	yVHVNAtyVNVKCVA	Y1354-p	yVHVNATyVNVKCVA	Y1357	YVHVNATYVNVKCVA
7	116	Y1365-p	NVKCVAPyPsLLssE	Y1363-p	NVKCVAPyPSLLPSQ	Y1366	NVKCVAPYPSLLPSQ
0	8	S1367-p	KCVAPyPsLLssEDN	S1365	KCVAPyPSLLPSQDN	S1368	KCVAPYPSLLPSQDN

Data are from PhosphoSitePlus (www.phosphosite.org) only sites with 5 or more references are reported. SS, site-specific studies; MS, mass spectrometry in discovery mode; * autocatalysis.

The best-characterized MET signal transducers and their role in signaling are summarized below:

**GRB2**, an adaptor which binds Y_1356_ inserted in a YVNV motif [72]. Through its SRC homology region 3 (SH3), GRB2 recruits to the receptor the Ras activator SOS, promoting the MAPK signaling cascade. The GRB2 SH3 also binds a GAB1 sequence rich in prolines, promoting the association to the receptor of GAB1, which is in turn heavily phosphorylated [77].

**GAB1**, a member of IRS family of adaptors, sharing an amino-terminal pleckstrin homology (PH) domain that controls subcellular localization to areas of cell–cell contacts. It is recruited to MET through GRB2, but also contains a MET binding domain [78]. GAB1 presents multiple tyrosine residues that act to further recruit SH2 or phospho-tyrosine binding (PTB) domain-containing substrates [79]. Indeed GAB1 substantially contributes to the recruitment to MET of:

- PI3K, which binds GAB1 Y_447_, Y_472_ and Y_589_ [80];
- PLCγ, which binds GAB1 Y_307_, Y_373_, Y_407_ [81];
- NCK, an adaptor molecule, which binds GAB1 Y_407_;
- CRK-I and CRK-II, a family of adaptors [82]. CRK recruits via its first SH3 domain several downstream signal transducers such as C3G, an activator of the small GTPase Rap1 [83] resulting essential for HGF induced motility [84];
- SHP-2, which binds GAB1 Y_637_. SHP-2 acts as a tyrosine phosphatase but also as an adaptor to sustain MAPK signaling [85] and decrease Rho promoted stress fibers [86], resulting essential for motility and morphogenesis [82].

**NCK**, an adaptor protein, which contains three SH3 domains followed by one SH2 domain. HGF promotes Nck activation and its co-precipitation with PLCγ [87,88]. NCK also regulates actin polymerization and remodeling by recruiting N-WASP and the WAVE complex [89], promoting dorsal ruffles formation [90].

**SHC**, an SH2 containing adaptor that, after stimulation of the HGF receptor, is phosphorylated on Y_317_, generating a further high affinity binding site for GRB2 and triggering the Ras pathway [73].

**PI3K**, the class I phosphatidyl inositol 3-kinase associates via the p85 subunit to GAB1 but also bind directly to the receptor. Upon receptor recruitment to the receptor PI3K is tyrosine phosphorylated and activated [91,92,93]. PI3K activity is critical for MET-mediated chemotaxis and tubulogenesis and less for mitogenesis [94,95]. PI3K also coordinates survival and metabolism via phosphatidylinositol 3,4,5-trisphosphate dependent recruitment and activation of PH containing proteins such as the kinases PDK and AKT.

**PLCγ**, a phosphoinositide-specific phospholipase, which associates to the receptor and is tyrosine phosphorylated and activated. PLCγ promotes an early peak of inositol 1,4,5-trisphosphate (IP3) and 1,2-diacylglycerol (DG) release [92], while at later times the DG is sustained by phosphatidylcholine-specific PLC activity [96]. PLCγ activity leads to IP3 mediated release of Ca^2+^ from intracellular stores within seconds and induces Ca^2+^ oscillation peaking at 2 to 5 hours [97]. The parallel accumulation of Ca^2+^ and DG sustains classical protein kinase C (PKC) [98], which are not required for MAPK activation but collaborates with p38 and p42/p44 MAPKs in HGF-induced proliferation [99].

HGF also activated phospholipase D (**PLD**) in a PKC dependent way. PLD hydrolyzes phosphatidylcholine to choline and phosphatidic acid (PA), which is further metabolized to DG by PA phosphohydrolase (PAP). The PLD-PAP pathways quantitatively contributes to DG accumulation and to the expressions of JUN and FOS transcription factors [100].

**SRC**, the proto-oncogene tyrosine-protein kinase SRC is the prototype of a family of closely related tyrosine kinases among which SRC [101] and FYN [92] bind to MET and are activated upon HGF stimulation. SRC contributes to GAB1 phosphorylation, and is essential for HGF induced motility and transformation but dispensable for proliferation [102]. SRC activation by MET promotes tyrosine phosphorylation of several downstream proteins not directly associated to the receptor such as:

- FAK, the focal adhesion kinase, which lies at the crossroad between integrin and growth factor signaling. The FAK Y_194_ is also directly phosphorylated by MET contributing to activation [103]. Once activated FAK induces downstream GRB2 binding and MAPK signaling, critically controlling the cytoskeleton [104];
- αDGK (Diacylglycerol kinase alpha), which phosphorylates DG to PA. αDGK is phosphorylated by SRC on Y_335_ and its activity is crucial for HGF-induced cell motility by promoting PA production at ruffling sites. This drives local recruitment of PA binding proteins involved in migration such as the Rho GDP-dissociation inhibitor (RhoGDI) and atypical PKC [105,106,107,108] as well as integrin recycling such as the Rab11 interactor RCP [109].

**STAT3**, a transcription factor, which upon HGF treatment is recruited to MET, tyrosine phosphorylated and translocates in the nucleus within in hours. STAT3 directly couples MET signaling to the transcription program required for tubule formation *in vitro*, without affecting either HGF-induced scattering or growth [110,111].

**SHIP-1/2**, the SH2 domain-containing inositol 5-phosphatases 1 and 2. SHIP-1/2 binds at Y_1356_ and positively affects lamellipodia extension and tubulogenesis [112,113].

Phospholipase A2 (**PLA2**), is not complexed to the receptor but tyrosine phosphorylated by MET and serine phosphorylated by MAPK. PLA2 triggers arachidonic acid release by membrane phospholipids and is functionally coupled with the HGF triggered activation of the COX-2 transcription through the MAPK signaling pathway [114]. The production of arachidonic acid derived mediators by COX-2 amplifies MET signaling and putatively coordinates epithelial sheet responses in a paracrine fashion [115].

Consistently with the pathways described, a global analysis of MET signaling in small cell lung cancer using phospho-arrays identified as positively regulated HGF targets:

- MET (Y_1003_/Y_1230_/Y_1234_/Y_1235_);
- phosphoproteins that regulate transcriptional control: STAT3 (S_727_) and CREB (S_133_);
- cell cycle G1/S checkpoint: RB (S612), RB1 (S780);
- cell survival and apoptosis: AKT1 (S473/T308), JNK (T183/Y185);
- cell proliferation and differentiation: MEK1/2 (S221/S225), ERK1/2 (T185/Y187), ERK1/2 (T202/Y204);
- stress and inflammatory response to cytokines and growth factors: MEK3/6 (S189/S207), p38α (T180/Y182); JNK (T183/Y185);
- Cytoskeletal functions: FAK (Y_576_/S_722_/S_910_), adducin-α (S_724_) and adducin-γ (S662).

In the same study a modest inhibition of HGF-induced phosphorylation in the following phosphoproteins was detected: PKCα (S_657_), PKCα/β (T_368_/_641_), and PKCδ (T_505_); the anti-proliferative and pro-apoptotic PKR (T_451_) and the cell cycle checkpoint regulator CDK1 (T_14_/Y_15_) [116].

### 3.4. Biological Effects of MET Triggering

The interest on HGF–MET signaling has been boosted by its intriguing ability to conjugate in epithelial cells the typical growth factor capability of promoting proliferation and survival with a very strong motogenic activity. The coupling of these two activities in a single “invasive growth program” points to a key role in both embryonic development and tumor metastatization [21]. Indeed a global analysis of HGF/MET dependent transcriptome indicates that targets of the MET pathway included genes involved in the regulation of cell motility, cytoskeletal organization, angiogenesis and oxidative stress responses [117].

Those activities are very reminiscent of epithelial to mesenchymal transition (EMT), a complex program that during embryogenesis enables epithelial cells to acquire mesenchymal migratory phenotype, populate and establish tissues in distant body regions. EMT is also exploited by tumor cell for metastatic dissemination and chemoresistance [118]. Indeed HGF induces EMT master genes such as Snail and Slug via the early growth response protein 1 (Egr1) [119,120]. Furthermore, migration and repopulation of distant sites are reminiscent of a stem phenotype, indeed HGF triggering of MET induces a stem-like phenotype in human prostate cancer cells with a stem-like signature of gene expression and markers of stemness [121]. In untransformed stem cells, HGF and MET are involved in the exit from quiescence and pre-activation [122].

**Figure 2:**
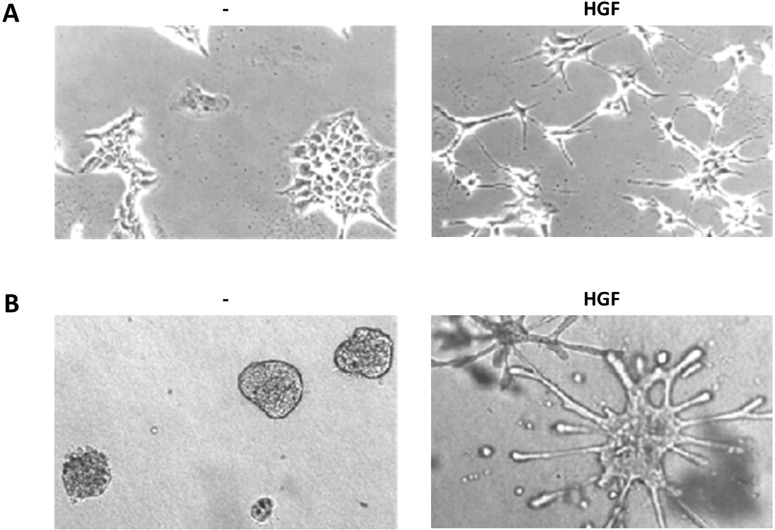
HGF biological assays. (**a**) Scatter assay. Mouse embryonic liver derived cells (MLP-29, [123]) cultured on plastic in presence or absence of HGF (5 U/mL, 24 h); (**b**) Branching morphogenesis assay. MLP-29 cells cultured in collagen gels in presence or absence of HGF (20 U/mL, 7 days).

The HGF peculiar capabilities have been characterized in typical *in vitro* assays, widely used to study HGF and MET activity and signaling, which are described below together with the main signaling pathways involved (Figure 2).

#### 3.4.1. Scattering

Serum-starved untransformed epithelial cells (typically MDCK, Madin-Darby canine kidney epithelial cells [2]) in 2D cultures grow as monolayers with junctional complexes reminiscent of epithelial sheets. HGF stimulation induces a rapid loss of actin stress fibers, which are replaced by smaller peripheral actin filaments, and extensive membrane ruffling. After 4 h of HGF treatment become apparent a loss of junctional complexes (comprising E-cadherin, desmoplakins and the tight junction protein ZO-1) and an increased tyrosine phosphorylation of β-catenin [124]. At the same time the cells loose the cuboidal epithelial shape and the apico-basal polarity to spread and elongate in a fibroblast-like morphology with leading edge-trailing edge polarity, morphologically reproducing EMT [71,125].

HGF-induced cell–cell dissociation and the following dispersion are dependent on Ras and on the downstream activation of both MAPK and PI3K [71,126,127,128,129]. Prolonged activation of Ras-MAPK signaling downstream of HGF is known to promote cell migration, paxillin phosphorylation [127,130,131,132] and expression of matrix metalloproteinase (MMP) 9 via the transcription factors Elk-1 and FOS [133]. Concomitantly the PI3K-AKT-S6K pathway stimulates the expression and proteolytic activity of MMP-9 and matrix invasion [134]. HGF also causes tyrosine phosphorylation and redistribution of β-catenin in the hepatocytes and this effect is attributable to subcellular association of MET and β-catenin. HGF treatment of mouse mammary cells also leads to a transient decrease in GSK3 kinase activity and a parallel increase in the nuclear accumulation of β-catenin and activation of a LEF responsive reporter gene. Part of the EMT program evoked by HGF might be attributable to nuclear β-catenin and the resulting target gene expression [135,136].

In HGF treated epithelial MDCK colonies, MET is transcytosed from the basolateral membrane on Rab4 endosomes, to the apical surface where MET, as well as the MET substrate and scaffold protein, GAB1, localizes and signals at the dorsal ruffles [137]. As dorsal ruffles collapse, MET is internalized into EEA1- and Rab5-positive endosomes and is targeted for degradation through delivery to an Hrs-positive sorting compartment (see below).

The considerable HGF-induced reorganization of the actin cytoskeleton involved in scattering is mediated by the Rho family GTPases downstream to both Ras and PI3K [128]. Indeed HGF promotes activation of Rac1, RhoA and Cdc42 concomitant with the formation of filopodia and lamellipodia [138,139]. Cdc42 and Rac1 activities are required for HGF-induced cell–cell dissociation in MDCK cells but also for inducing ruffling and spreading by promoting activation of the Cdc42/Rac-regulated p21-activated kinase PAK [126,139]. The activation of Rac and Cdc42 is long lasting (till 24 h), consistent with a persistent polarized and migratory phenotype [140]. According with a starter role of Rho in ruffling [141,142] and with the observed prolonged decrease in stress fibers and increased spreading, a transient RhoA activation is observed in the first minutes of stimulation [143,144]. Indeed, inhibition of RhoA blocks HGF-induced cell scattering [128,142] and inhibition of the Rho-dependent kinase (ROCK) results in a reduction in HGF-induced membrane protrusion, reduction of dissociation and impaired motility with cell assuming a typical shape with elongated tails due to an impairment of tail retraction [138,139,145].

In connection with the actin cytoskeleton, there is a remodeling of adhesion sites, with a reduction in large paxillin-associated substratum adhesions particularly in areas of active membrane protrusion, where they are replaced by small peripheral focal contacts, which continuously disassemble or mature to focal adhesions during cell locomotion. Within minutes after exposure of HGF, FAK and paxillin become transiently phosphorylated in coincidence with the conversion to a motile phenotype [146,147]. HGF can also induce serine/threonine phosphorylation of paxillin most probably mediated directly by ERK, resulting in the recruitment and activation of FAK and subsequent enhancement of cell spreading and adhesion [130]. Rho and ROCK activity are required for the formation of mature focal adhesions by promoting cytoskeletal tension and stress fibers in response to HGF [139]. At the same time HGF promotes cell adhesion and invasiveness by increasing the avidity of integrins for their specific ligands in a PI3K dependent way [148,149].

#### 3.4.2. Branching Morphogenesis

When plated in 3D collagen rich reconstituted extracellular matrix, untransformed epithelial cells (also in this case mainly MDCK) forms hollow cysts with typical apico-basal polarity. In polarized epithelial cells MET asymmetrically distributes at the basolateral membranes reflecting association with cell to cell and cell to matrix contacts [150,151]. At the membrane MET partially colocalize with caveolin in detergent resistant membranes and exploit caveolin for signal amplification [152].

Upon HGF treatment, cysts undergo growth and extend in the matrix forming tubular structures with interconnected lumens, in a process reminiscent of the epithelial organogenic program. Early in tubule development, MDCK cells exhibit many features characteristic of EMT forming long, invasive cytoplasmic extensions. Extension formation requires PI3K activity, whereas ROCK controls their number and length [153].

Extensions next proliferate and arrange in rows from one to three cells long, showing elongated cells which invade the matrix. While cells in the monolayer divide with their spindle axis parallel to the monolayer, HGF dislodge the spindle axis so that one of the daughter cells can apparently leave the monolayer to initiate a chain [153]. Those cells loose apico-basal polarity and gain leading edge-trailing edge polarity but maintain some intercellular adhesion representing a partial and transient EMT. Indeed cells in chains redifferentiate [154], loose their mesenchymal qualities and form multilayered cords by expanding regions of cell-cell contact and reestablishing a cuboidal shape. Nascent lumens and incomplete apical and basolateral domains appear. Eventually, cords mature into tubules through formation of a single continuous lumen and coordinated apico-basal polarization of individual cells.

Activation of ERK is necessary and sufficient for the initial step, during which cells depolarize and migrate, while becomes dispensable for the latter stage, during which cells repolarize and differentiate [155]. Conversely MMPs are essential for the late re-differentiation stage of tubulogenesis [155]. MMPs represent a class of HGF effectors positively regulated by HGF and involved in tubulogenesis and scattering. Indeed in glioblastoma cells and endothelial cells prolonged induction of ERK signaling by HGF promotes expression and secretion of MMP-2 and upregulation of MT1-MMP, a cell-surface activator of proMMP-2 [156,157]. Similarly in keratinocytes HGF induced scattering but not proliferation requires MMP-9 induction due to sustained activation of ERK kinases [158].

An emerging player in tubulogenesis is ARF6 which is necessary and sufficient to initiate tubule extension by both regulating the subcellular distribution of Rac1 to tubule extensions but also by inducing ERK mediated expression of the receptor for urokinase type plasminogen activator [159].

#### 3.4.3. Balance between Proliferation and Apoptosis

The invasive growth program induced by HGF critically requires two other key features to succeed: increased resistance to apoptosis and enhanced proliferation.

The increased resistance to apoptosis is a typical feature of EMT and allows the invading epithelial cells to survive without the physiological survival signals provided by cell–cell and cell–matrix contacts, avoiding death for anoikia. HGF triggering of MET protects cells from apoptosis by using mainly the PI3K/Akt and, to a lesser extent, the MAPK pathways [160,161]. The PI3K-AKT pathway triggers Bad phosphorylation, thereby inactivating this pro-apoptotic protein, while simultaneously inducing expression of anti-apoptotic proteins such as Bcl-xL and Mcl-1 [162,163]. Furthermore PI3K/Akt, via the mTOR kinase, promote the translation and nuclear import of Mdm2, which inhibits TP53 activity both *in vitro* and *in vivo* [164]. MET induced resistance to apoptosis also allows tumor cells to resist to conditions that they face during tumor progression, *i.e.*, nutrient deprivation or substrate detachment as well as chemotherapeutic treatment. HGF-driven survival of carcinoma requires the engagement of the PI3K/Akt/mTOR/S6K and ERK/MAPK transduction pathways, cooperatively preventing stress induced p38 activity [165].

Proliferation is also a typical response of many cell types to HGF triggering with an early peak of JUN and FOS transcription and activation (0.5–3 h) followed by Myc expression (6–8 h) and increased expression of cyclins A, B, D, and E (12 h) [166,167]. Thus, HGF promotes both increased transcription of AP-1 (FOS/JUN complex) and Myc early response genes [87] but also FOS mRNA translation via the PI3K/mTOR/4E-BP1 [168]. Nuclear factor kappa-B (NF-κB) is a multivalent transcription factor, which potentially controls the apoptosis/proliferation balance depending on the cellular context. HGF promotes NF-κB activation via both the PI3K/AKT and the Ras/MAPK pathway, mediating a survival signal [169] but also proliferation and morphogenesis [170].

Surprisingly HGF is a potent mitogen for a variety of cell types, but it is also known as an anti-mitogenic factor for several types of tumor cell lines. In HepG2 the high intensity ERK signal causes cell cycle arrest at G1 increasing the Cdk inhibitor p16-INK4a [171] and p21 [172], which mediates growth inhibition in the presence of HGF.

### 3.5. Negative Regulation of MET Signaling

As tyrosine phosphorylation is the key event in MET signaling, several researchers have worked to identify the tyrosine phosphatases (PTP) terminating receptor activation. PTP is a large family of more than 100 genes in humans comprising both soluble enzymes reversibly associating to protein targets and trans-membrane enzymes regulated by both extracellular and intracellular cues. MET phosphorylated in the activation loop is a substrate for cytosolic phosphatases such as PTP-1B and T-cell phosphatase [173]. Also PTP-S binds specifically to the juxtamembrane region of the activated receptor [174]. MET is similarly targeted by receptor-type protein tyrosine phosphatases such as: (1) RPTP-β, which dephosphorylates MET Y_1356_ and impairs MET tumorigenic activity [175,176]; (2) LAR, which counteracts MET auto-phosphorylation, as well as downstream MAPK and PI3K activation mediating contact inhibition [177,178]; (3) CD148, which dephosphorylates the tyrosines recruiting downstream effectors as well as the associated signal transducers GAB1 and p120 catenin [179].

An independent way of regulating MET activity resides in the juxtamembrane region which contains a cluster of serine/threonine phosphorylation sites (Figure 1 and Table 1). The best characterized of these sites is S_985_ which is phosphorylated by PKC-δ and -ε and dephosphorylated by protein phosphatase 2A [180]. S_985_ phosphorylation event inhibits the ligand-induced tyrosine auto-phosphorylation of the receptor and the receptor tyrosine kinase activity on exogenous substrates [181]. The relevance of such region is underscored by the existence of a splicing isoform of MET without such region and endowed with enhanced transforming ability [41,182].

MET and its associated proteins undergo ligand induced internalization that allows efficient signaling from endosomes but also couple with degradation of both receptor and ligand [183]. Indeed, upon ligand binding, the MET-HGF complex is rapidly internalized and MET become polyubiquitinated by Cbl. Cbl is an E3 ubiquitin ligase that associate in the juxtamembrane region of MET upon Y_1003_ phosphorylation and is phosphorylated upon HGF stimulation. A complex comprising Cbl, the adaptor CIN85 and endophilin promotes the import of MET in multivescicolar bodies trough clatrin coated pits. In this process Cbl is not a mere negative regulator of MET as it also acts as a scaffold for effectors recruitment in endosomes [184]. From the multivescicular body the receptor/ligand complex eventually recycles to the cell surface or is degraded in the lysosomes [185,186]. To the control of ubiquitinated-MET recycling cooperates Hrs, an early endosomal protein that is rapidly tyrosine-phosphorylated in cells stimulated with growth factors. Hrs couples with Stam to constitute the ESCRT complex that controls the initial selection of ubiquitinated proteins into clathrin-coated microdomains of early endosomes. Hrs also promotes receptor traffic toward multivesicular bodies/lysosomes by interacting with sorting nexin1 [187,188]. Conversely, proteasome activity is required for MET internalization and only indirectly for its degradation [189].

## 4. Signaling Integration by Met Multi-Receptor Complexes

MET has shown the remarkable property to associate with other signaling molecules to form complexes with several other receptors, effectively working as platforms for signal integration and amplification.

MET selectively associates with α6β4 integrin at the plasma membrane contributing to promote invasive growth, independently from laminin binding. CD151, a transmembrane protein of the tetraspanin family is a critical components of the complexes between MET and β4 integrin [190]. Following MET activation, α6β4 is tyrosine phosphorylated and combines with SHC, PI3K and SRC, generating an additional signaling platform that potentiates HGF-triggered activation of Ras- and PI3K-dependent pathways [191], promoting invasion [192] and anchorage independent growth [193]. This association between MET and integrins seems not to be an isolated instance as fibronectin binding to α5β1 integrin leads to a direct association of α5-integrin with MET, activating it in a HGF independent manner and promoting activation of SRC and FAK [194].

Semaphorins are cell surface and soluble signals that control directed migration and axonal guidance by binding to plexins receptors. The SEMA domain in the extracellular part of MET has strong homology to both plexins and semaphorins [195], suggesting a possible interaction between the two receptor families. Indeed Plexin B1 and MET associate in a complex and activation of Plexin B1 by SEMA 4D stimulates the tyrosine kinase activity of METMET, resulting in tyrosine phosphorylation of both receptors and downstream signaling [196,197,198]. Similarly to Plexins also Neuropilins acts as receptors for semaphorins and Neuropilin-1 associates with MET promoting its HGF induced activation and cell invasiveness [199]. Furthermore both Neuropilin-1 and Neuropilin-2 bind HGF, potentially acting as MET co-receptors [200].

A CD44 isoform containing variant exon v6 sequences (CD44v6) is strictly required for MET activation by HGF/SF, independently from HS modification of CD44. Autophosphorylation of MET requires the formation of a complex formed by HGF, MET and CD44v6. In this complex CD44v6 cytoplasmic tail presents binding motif for ezrin, radixin and moesin (ERM) proteins [201], which are phosphorylated by MET [202]. In this complex association of ERM proteins with CD44v6 and their link to the actin cytoskeleton is absolutely required to mediate the HGF-dependent activation of Ras, indicating a scaffolding function of cytoskeleton in HGF signaling [203]. CD44v6 mediated ERM binding to MET also participates to HGF induced receptor internalization [204]. Of note, CD44v6 is a marker of cancer stem cells, functionally cooperating with MET to promote PI3K dependent metastatic growth [205].

Those studies are just examples of how MET cooperates with a variety of other receptor for signaling integration. Of particular relevance for metabolism is the cooperation with insulin signaling. Indeed, in hepatic cells, MET form a complex with insulin receptor which respond to HGF triggering with trans-phosphorylation of the insulin receptor, recruitment of IRS1/2, stimulation of hepatic glucose uptake and suppression of hepatic glucose output [206]. MET also promotes PI3K dependent glucose uptake and glucose utilization from adipocytes [207], skeletal muscle cells [208] and participates to glucose homeostasis *in vivo* [206]. This is not an isolated example as MET can also be trans-activated and co-immunoprecipitated by other growth factor receptors such as RON [209,210], EGFR [211], HER2, HER3, and RET [212]. Hetrodimerization between growth factor receptors offers a platform to support signal integration, however in few cases the molecular bases of clustering and their relevance for signaling were not fully understood.

## 5. Conclusions

Thirty years of studies have explored in depth the signaling pathways promoted by the two simple tyrosines in MET cytoplasmic tail. However, the multiple intersections of those pathways as well as the interactions of MET with other transmembrane transducers point to the necessity of switching the approach from a signaling pathway oriented view to a network approach. In such framework a quantitative evaluation of signaling coupled to mathematical modeling could contribute to clarify how the cells integrates MET signaling in its biological context.

Indeed the fascinating question that drove the HGF-MET research still persists: How does a single factor—single receptor couple exerts a pleiotropy of biological effects in a context dependent manner?

## Abbreviations

**Short name (used in the manuscript)**	**Full name (Uniprot)**
**HGF**	Hepatocyte growth factor
**MET**	Hepatocyte growth factor receptor
**MSP**	Hepatocyte growth factor-like protein
**RON**	Macrophage-stimulating protein receptor
**CD44v3**	CD44 antigen including variant exon 3
**CD44v6**	CD44 antigen including variant exon 6
**InlB**	Internalin B
**GRB2**	growth factor receptor-bound protein 2
**PLCγ**	1-phosphatidylinositol 4,5-bisphosphate phosphodiesterase gamma
**PI3K**	Phosphatidylinositol 4,5-bisphosphate 3-kinase
**SHP-2**	tyrosine-protein phosphatase non-receptor type 11
**SHC**	SHC-transforming protein
**GAB1**	GRB2-associated binding protein
**MAPK**	mitogen-activated protein kinase
**SOS**	Son of sevenless homolog
**NCK**	cytoplasmic protein NCK
**CRK-I and CRK-II**	adapter molecule crk
**C3G**	Rap guanine nucleotide exchange factor 1
**N-WASP**	Neural Wiskott-Aldrich syndrome protein
**PKD**	Serine/threonine-protein kinase D
**AKT**	RAC-alpha serine/threonine-protein kinase
**PKC**	Protein kinase C
**PLD**	Phospholipase D
**PAP**	Phosphatidic acid phosphohydrolase
**JUN**	Transcription factor AP-1
**FOS**	Proto-oncogene c-Fos
**SRC**	proto-oncogene tyrosine-protein kinase SRC
**FYN**	Tyrosine-protein kinase Fyn
**FAK**	Focal adhesion kinase
**αDGK**	Diacylglycerol kinase alpha
**RhoGDI**	Rho GDP-dissociation inhibitor
**RCP**	Rab11 family-interacting protein 1
**STAT3**	Signal transducer and activator of transcription 3
**SHIP-1**	SH2 domain-containing inositol 5-phosphatases 1
**SHIP-2**	SH2 domain-containing inositol 5-phosphatases 2
**PLA2**	Phospholipase A2
**COX-2**	Prostaglandin G/H synthase 2
**CREB**	Cyclic AMP-responsive element-binding protein
**RB**	Retinoblastoma-associated protein
**JNK**	Mitogen-activated protein kinase 8
**MEK-1**	Dual specificity mitogen-activated protein kinase kinase 1
**MEK-2**	Dual specificity mitogen-activated protein kinase kinase 2
**ERK1**	Mitogen-activated protein kinase 3
**ERK2**	Mitogen-activated protein kinase 1
**MEK3**	Dual specificity mitogen-activated protein kinase kinase 3
**MEK6**	Dual specificity mitogen-activated protein kinase kinase 6
**PKR**	Protein kinase R
**p38α**	Mitogen-activated protein kinase 14
**CDK1**	Cyclin-dependent kinase 1
**Snail**	Zinc finger protein SNAI1
**Slug**	Zinc finger protein SNAI2
**Egr1**	early growth response protein 1
**Elk-1**	ETS domain-containing protein Elk-1
**S6K**	Ribosomal protein S6 kinase beta
**GSK3**	Glycogen synthase kinase-3
**Lef**	Lymphoid enhancer-binding factor
**PAK**	Serine/threonine-protein kinase PAK 1
**ROCK**	Rho-associated protein kinase
**MT1-MMP**	Matrix metalloproteinase-14
**BAD**	Bcl2-associated agonist of cell death
**Bcl-xL**	Bcl-2-like protein 1 (long isoform)
**Mcl-1**	Induced myeloid leukemia cell differentiation protein Mcl-1
**mTOR**	Serine/threonine-protein kinase mTOR
**Mdm2**	E3 ubiquitin-protein ligase Mdm2
**TP53**	Cellular tumor antigen p53
**MYC**	Myc proto-oncogene protein
**4E-BP1**	Eukaryotic translation initiation factor 4E-binding protein 1
**p16-INK4a**	Cyclin-dependent kinase inhibitor 2A
**p21**	Cyclin-dependent kinase inhibitor 1
**PTP-1B**	Tyrosine-protein phosphatase non-receptor type 1
**RPTP-β**	Receptor-type tyrosine-protein phosphatase B
**LAR**	Receptor-type tyrosine-protein phosphatase F
**CD148**	Receptor-type tyrosine-protein phosphatase eta
**Cbl**	E3 ubiquitin-protein ligase CBL
**CIN85**	SH3 domain-containing kinase-binding protein 1
**Hrs**	Hepatocyte growth factor-regulated tyrosine kinase substrate
**Stam**	Signal transducing adapter molecule
**IRS1**	Insulin receptor substrate 1
**IRS2**	Insulin receptor substrate 2
**EGFR**	Epidermal growth factor receptor
**HER2**	Receptor tyrosine-protein kinase erbB-2
**HER3**	Receptor tyrosine-protein kinase erbB-3
**RET**	Proto-oncogene tyrosine-protein kinase receptor Ret
